# Mr. Hervey Coke Parke

**Published:** 1899-03

**Authors:** 


Mr. Hervey Coke Parke, the senior member of the house of Parke, Davis & Co., of Detroit, died February 8, 1899, at this winter home in southern California, whither he had lately gone to escape the rigors of late winter and early spring that usually prevail in this latitude. Mr. Parke had attained the ripe age of 72 years, but was an active man of affairs to the very last. In his youth he attended a high school in Buffalo and completed his studies at Hobart College. He established the house with which his name has been so prominently associated in 1866, and in 1875 it was incorporated with a paid up capital of $100,000, which was subsequently increased to $1,200,000. This eminently successful house owes its prosperity in no small degree to the business sagacity and sterling integrity of Mr. Parke, who had been its president since 1875. He was a member of St. Johns Church, Detroit, where for fifteen years he had been a vestryman, and from which his funeral was held, February 15, 1899.

